# Brain MRI correlations with disease burden and biomarkers in Fabry disease

**DOI:** 10.1007/s00415-023-11826-8

**Published:** 2023-06-25

**Authors:** Yawen Zhao, Ying Zhu, Fan Li, Yunchuang Sun, Wei Ma, Yuan Wu, Wei Zhang, Zhaoxia Wang, Yun Yuan, Yining Huang

**Affiliations:** 1 Department of Neurology, Peking University First Hospital, Xishiku Street, West District, Beijing, 100034 China; 2 Department of Medical Iconography, Peking University First Hospital, Beijing, China; 3 Department of Cardiology, Peking University First Hospital, Beijing, China; 4 Department of Ophtalmology, Peking University First Hospital, Beijing, China; 5 Beijing Key Laboratory of Neurovascular Diseases, Beijing, China

**Keywords:** Fabry disease, Cerebral small vessel disease, White matter hyperintensity, Mainz Severity Score Index, Globotriaosylsphingosine

## Abstract

**Objective:**

To quantitatively evaluate cerebral small vessel disease (CSVD) in brain magnetic resonance imaging (MRI) and its correlation with disease burden and markers in Fabry disease, a rare X-linked lysosomal storage disease.

**Methods:**

We collected brain MRI data from seventy-one Chinese patients with Fabry disease. CSVD was evaluated using an age-related white matter change rating scale, Fazekas scale, enlarged perivascular spaces grading scale, lacunar infarction scale, Microbleed Anatomical Rating Scale, global cortical atrophy scale, and small-vessel disease score. Factors associated with MRI lesions, including sex, clinical subtype, disease severity, disease burden, genotype, and biomarkers, were also analyzed.

**Results:**

Of 71 patients, 16 (22.5%) experienced ischemic stroke. The incidences of lacunar infarctions, white matter hyperintensities, and cerebral microbleeds were 55%, 62%, and 33%, respectively. The abnormal MRI group had later disease onset, longer disease duration, and a higher Mainz Severity Score Index (*p* < 0.05) than the normal MRI group. Patients with more severe clinical phenotypes also had higher CVSD-related scores. Sex and *GLA* mutational type were not closely associated with brain MRI lesions. Of the disease markers, the Mainz Severity Score Index and plasma globotriaosylsphingosine (Lyso-Gb_3_) were closely correlated with the majority of the MRI scores, whereas α-galactosidase A activity was not.

**Conclusion:**

Brain MRI revealed progressive lacunar infarctions, white matter hyperintensities, and decreased brain volume in patients with Fabry disease. Brain MRI lesions were closely related to onset-age; disease duration, severity, burden; and plasma Lyso-Gb_3_. However, they were not associated with sex, α-galactosidase A activity, or *GLA* mutation type.

**Supplementary Information:**

The online version contains supplementary material available at 10.1007/s00415-023-11826-8.

## Introduction

Anderson–Fabry disease, also known as Fabry disease (OMIM301500), is a rare X-linked lysosomal storage disease that is caused by mutations in the *GLA* gene, located on Xq21.33–Xq22. *GLA* mutations decrease the activity of α-galactosidase A (α-Gal A), which increases the storage of enzymatic substrates as globotriaosylceramide. Globotriaosylceramide can deposit in many types of parenchymal cells, leading to the dysfunction of multiple organs including the heart, kidney, brain, lung, skin, eyes, and gastrointestinal tract [1]. Plasma globotriaosylsphingosine (Lyso-Gb_3_) is a helpful biomarker for the diagnosis and therapeutic monitoring of patients with Fabry disease [2]. The vital organ capacity insufficiencies of the kidney, heart, and brain are the main cause of a shortened lifespan in Fabry disease [1]. Fabry disease also plays an important role in early-onset stroke; in a systematic review and meta-analysis of over 8000 stroke patients, Fabry disease explained 4% of cryptogenic and 1% to 2% of all-cause early-onset strokes [3]. Moreover, a high proportion of patients with Fabry disease have unlocalized central nervous system (CNS) and neuropsychiatric symptoms [4].

Fabry disease is a hereditary cerebral small vessel disease (CSVD); brain MRI of patients shows a relatively high percentage of lacunar infarcts, white matter hyperintensities (WMH), enlarged perivascular spaces (EPVS), and cerebral microbleeds (CMB), with a relatively low incidence of territory infarcts [5, 6]. However, the detailed distribution and location of CVSD changes in Fabry disease have not yet been fully evaluated; Although WMH and CMB may be considered indicators of multiple organ involvement [7, 8], correlations between other CVSD imaging characteristics (such as lacunar infarction and EPVS) and disease severity have rarely been studied in Fabry disease. Thus, a topographical description of brain MRI will allow the CNS aspects of the disease to be more thoroughly understood and will provide diagnostic clues. Furthermore, correlations between CVSD changes in Fabry disease and other clinical aspects, such as disease burden evaluated using the Mainz Severity Score Index (MSSI) or the disease biomarkers α-galactosidase A (α-Gal A) and Lyso-Gb3, remain unclear. Therefore, the aims of the present study were twofold: (1) to summarize the CNS neuroimaging characteristics of Fabry disease, and (2) to evaluate the factors that influence cerebral small vessel lesions by evaluating the correlations of clinical aspects and biomarkers with MRI lesions.

## Materials and methods

### Patient data and subgroups

We enrolled 71 patients with Fabry disease (from 51 unrelated families) who underwent brain MRI in Peking University First Hospital, China, from January 2001 to September 2022. Diagnostic criteria were based on the typical clinical symptoms of Fabry disease, family history, and laboratory results (including *GLA* gene, α-Gal A activity, and plasma Lyso-Gb3) according to Chinese consensus in 2021 [9]. Mutations in the *GLA* gene were divided into truncated or non-truncated mutations based on mutation type. A pathological diagnosis (including kidney, nerve, or skin pathology) was also performed in some cases to reach a definite diagnosis. For this retrospective longitudinal cohort study, demographic information and detailed clinical and follow-up data from patients with one or more brain MRI on a 3 T scanner were extracted. The study was performed in accordance with the ethical standards of the relevant national and institutional committees on human experimentation and was approved by the Institutional Review Board of Peking University First Hospital (No. 2021 [061]; 2021[CJ1283]). Informed consent for all examinations was obtained from all patients or their guardians. The study protocol strictly followed the guidelines of the Declaration of Helsinki.

As well as conducting routine medical enquiries, we performed a thorough investigation of clinical profiles of the CNS, including stroke, dizziness, headache, tinnitus, hearing loss, vision loss, and other CNS-related symptoms. Risk factors of cerebrovascular disease were taken as hypertension, hyperlipidemia, hyperhomocysteinemia, diabetes, atrial fibrillation, and smoking history. After general and neurological examinations, the MSSI was performed to evaluate global clinical status [10]. Patients were divided into “classic” and “non-classic” disease types according to their clinical symptoms, α-Gal A activity (for male patients only), and Lyso-Gb3. [11] Patients were classified as having a severe phenotype if they experienced at least one of the following severe clinical events: (1) cardiac events (angina pectoris, arrhythmia, congestive heart failure, myocardial infarction, implantation of cardiac devices, or significant cardiac procedure); (2) renal events (first occurrence of either the initiation of chronic dialysis [> 40 days] or renal transplantation); (3) cerebrovascular events (hemorrhagic or ischemic stroke); and (4) death. Patients were classified as having a mild phenotype if they did not experience the aforementioned events [12].

### Brain MRI

Brain MRI data were obtained using 3 T scanners (3.0 T, GE 1.5 Sigma Twin Speed; GE Healthcare, Waukesha, WI, USA) with T1-weighted imaging, T2-weighted imaging, T2 fluid-attenuated inversion recovery, and diffusion-weighted imaging sequences in all patients. Susceptibility-weighted imaging sequences were performed in a subset of patients. Cerebral infarcts were classified as territory or lacunar infarcts. WMH were evaluated in five bilateral areas (frontal, parietal-occipital, and temporal lobes; basal ganglia; and infratentorial areas) using a scale of age-related white matter changes (ARWMC) [13]. Additionally, scans were coded for deep WMH using the Fazekas scale (from 0 to 3) [14]. EPVS were coded using the following scale in the basal ganglia, as applied to standard axial images: 0, no EPVS; 1, < 10 EPVS; 2, 11 to 20 EPVS; 3, 21 to 40 EPVS; and 4, > 40 EPVS. Centrum semiovale EPVS were coded as 0 (EPVS score of 0 or 1) or 1 (EPVS score of 2, 3, or 4). We then added together the basal ganglia and centrum semiovale EPVS scores to form a total EPVS score [15]. CMB were rated using the Microbleed Anatomical Rating Scale (MARS) [16]. Cerebral atrophy was evaluated using the global cortical atrophy (GCA) scale against a reference MRI brain template from neurologically normal subjects [17]. The total MRI burden of small-vessel disease score (SVDS) was rated on an ordinal scale from 0 to 4, and was calculated by counting the presence of each of the four MRI features of SVDs (WMH, EPVS, lacunar infarction, and CMB) [18]. Hyperintensity of the pulvinar was also specifically searched for in T1-weighted images. Scans were reviewed by two independent physicians, one of the readers (YW Zhao) had 10 years of experience in reading brain MRIs, and the other reader (Y Zhu) had over 20 years of experience reading brain MRIs. When there were conflicting data, the two readers reached an agreement after consultation.

Brain MRI of patients with Fabry disease were compared with brain MRI of age- and sex-matched neurologically normal controls. We also monitored cerebral small vessel lesion changes using rating scales; the annual change rates were defined as the changes in brain MRI scales/interval time between MRI examinations.

### Statistical analysis

IBM SPSS Statistics for Windows, version 25.0 (IBM Corp., Armonk, NY, USA) was used for all analyses. Data with a normal distribution are given as the mean ± standard deviation, and data with a non-normal distribution are given as the median (range or interquartile range). Single factor analyses were used to compare brain MRI changes between males/females, classic/non-classic groups, severe/mild phenotype groups, and different genotype groups. The Mann–Whitney U test was used to analyze continuous variables and the chi-squared test was used to analyze categorical variables. Multivariate logistic regression analysis was performed on related indicators of Fabry disease. Explanatory variables were selected using a liberal criterion (*p* < 0.10) for inclusion in the multivariate regression model. Pearson’s or Spearman’s methods were used for correlation analyses. A *p*-value of < 0.05 was considered significant.

## Results

### Clinical profiles

In all 71 patients (46 males and 25 females from 51 unrelated families) in our study, *GLA* sequencing of all seven exons and their flanking sequences revealed *GLA* gene mutations. Twenty-nine families (57%) had missense mutations, 19 families (37%) had frameshift or nonsense mutations, and three families (6%) had splicing mutations. Seventy patients underwent α-Gal A activity measurement; there was a marked decrease in activity (≤ 5% of normal range) in 39 males and a moderate decrease (6–24% of normal range) in the other seven males, whereas the 24 females had normal activity or different degrees of decrease (6–93% of normal range). Fifty-four patients underwent plasma Lyso-Gb3 measurement; the median (interquartile range) value was 26.61 (6.13, 85.71) ng/mL (Supplementary Table 1).

Of the 71 patients, one (P66) was asymptomatic; the onset age of the other 70 patients ranged from 5 to 70 years old (9.5 [7, 17.25]). Forty-five patients (63%) described CNS-related symptoms, which could be divided into two groups. In one group (acute episodes with neurological deficits), 16 patients (22.5%) experienced transient ischemic attack (1/16) or cerebral new infarctions (15/16), of whom six patients had recurrent stroke; age of first stroke ranged from 26 to 77 years old (40 [31.75, 50.75]). For the other group (chronic nonspecific neurological symptoms), patients described intermittent and fluctuating dizziness (55%), tinnitus (37%), hearing loss (25%), cognitive decline or memory loss (23%), headache (18%), depression (17%), and blurred vision (13%).

The risk factors of cerebrovascular disease in all 71 patients included smoking history (18%), hypertension (28%), hyperlipidemia (24%), hyperhomocysteinemia (20%), atrial fibrillation (4%), and diabetes (3%). The MSSI scores of 68 patients (P23, 24, and 57 were lost to follow-up) ranged from 0 to 48 (18 [10, 28]) (Supplementary Table 1).

In the present study, we also included 53 normal controls (37 males and 16 females). None of the normal controls had risk factors of cerebrovascular disease or described CNS-related clinical symptoms (Supplementary Table 2). There was no significant difference between controls and patients in terms of sex (*p* > 0.05).

### Brain MRI characteristics

#### Lesions and their locations

**Fig. 1:**
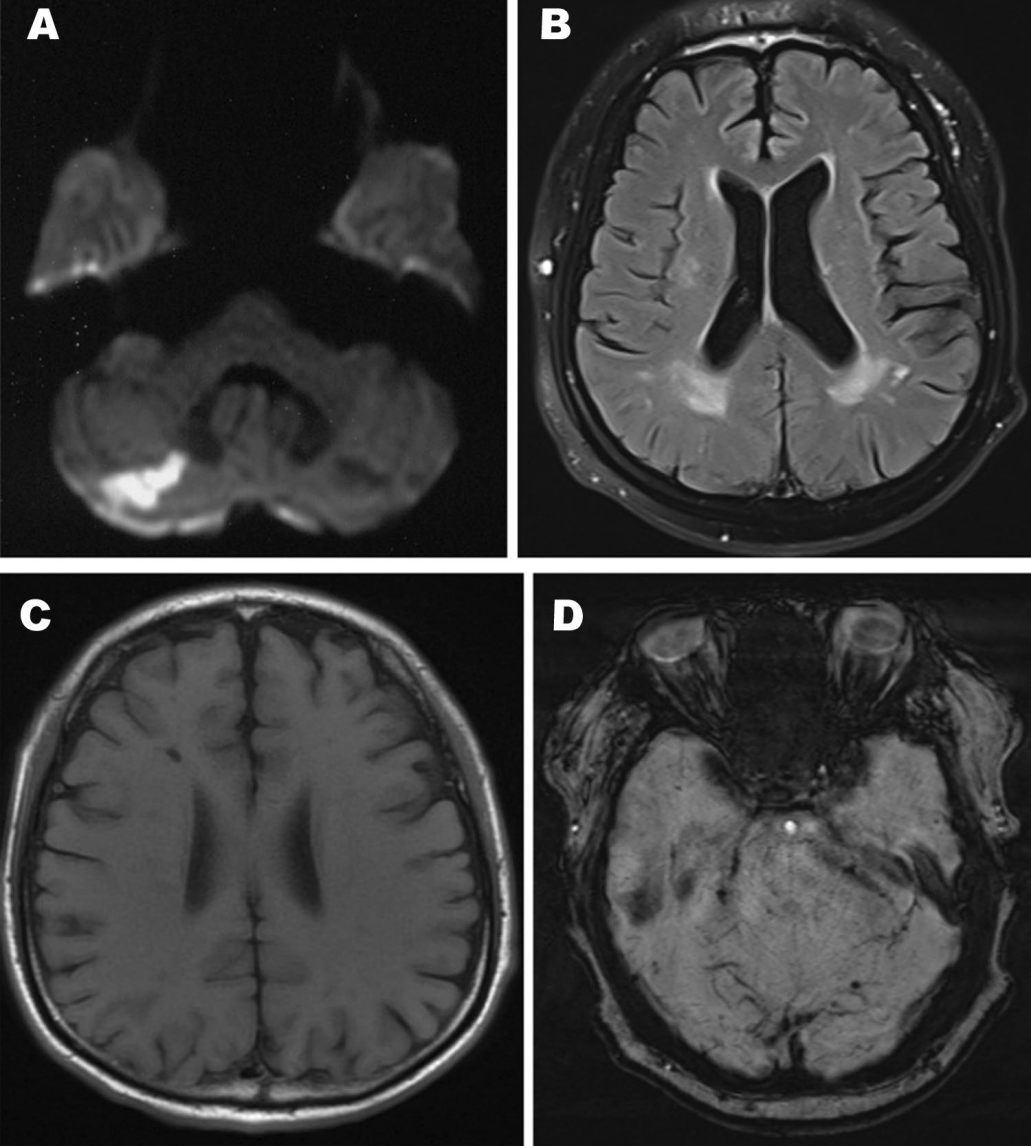
The brain MRI changes in Fabry disease. **A** DWI sequence showed recent cerebral infarctions in cerebellum. **B** T2 flair sequence showed white matter hyperintensity in parietal-occipital lobe and basal ganglia. **C** T1 sequence showed lacunar infarction in basal ganglia. **D** SWI sequence showed cerebromicrobleeds in infratentorial areas

**Fig. 2:**
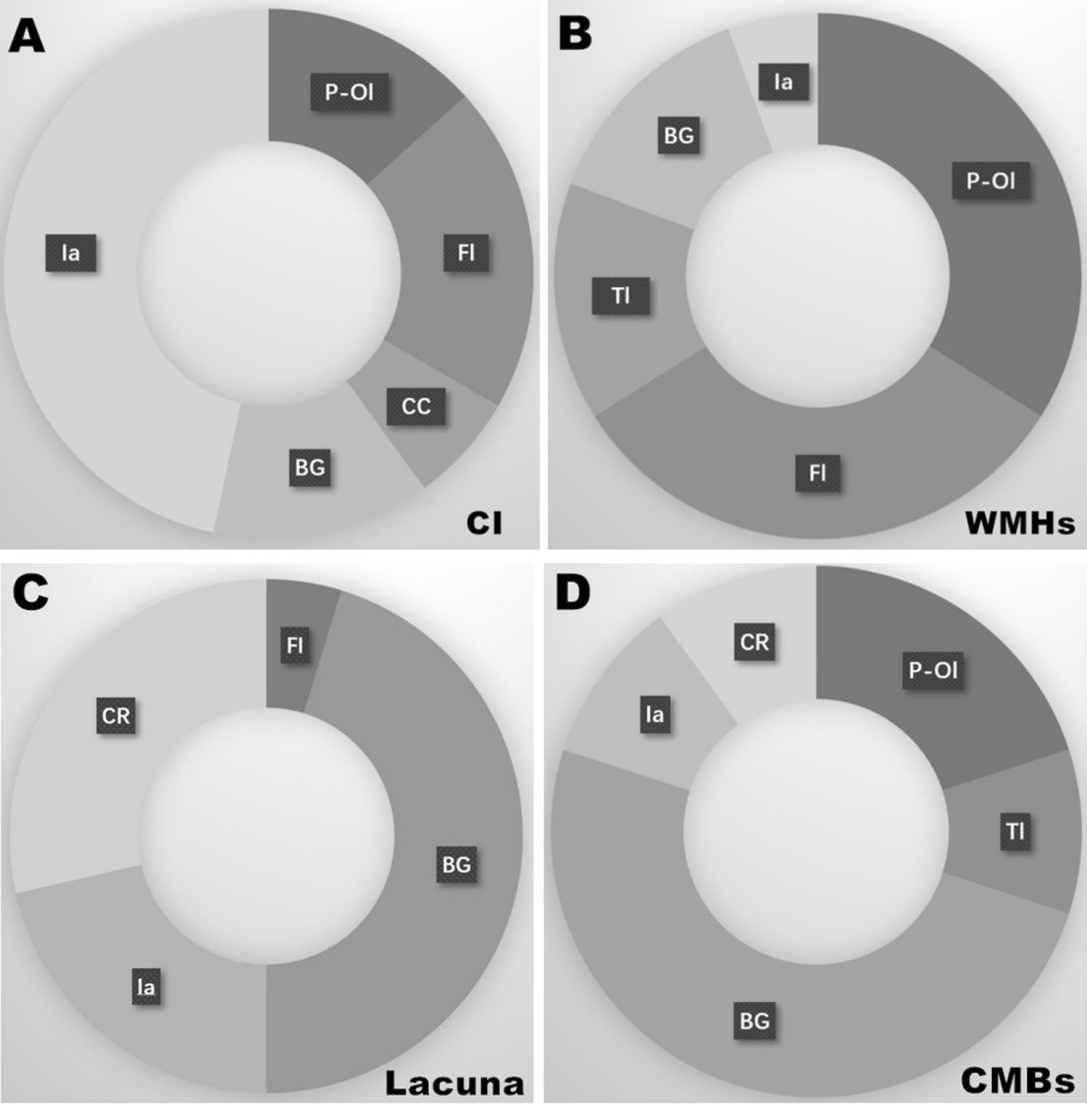
The Lesions and locations of CVSD imaging characteristics in Fabry disease. **A** Cerebral infarctions mostly located on infratentorial areas, frontal lobe and parietal-occipital lobe in Fabry disease. **B** White matter hyperintensity mostly located on parietal-occipital lobe, frontal lobe and temporal lobe in Fabry disease. **C** Lacunars infarction mostly located on basal ganglia, coronary radiata and infratentorial areas. **D** CMBs mostly located in basal ganglia. (*Ia* infratentorial areas; *FI* frontal lobe; *P-OI* parietal-occipital lobe; *BG* basal ganglia; *CC* corpus callosum; *TI* temporal lobe; *Ia* infratentorial areas; *CR* coronary radiata)

Age at the time of brain MRI in the 53 controls ranged from 7 to 77 years old (median 32 [25, 43] years), and in the 71 patients ranged from 7 to 77 years old (median 33 [25.5, 44] years). There was no significant difference in MRI examination age between controls and patients (*p* > 0.05). In the patient group, the interval between disease onset and MRI examination was 19 (10, 28) years. Overall, 44 patients (62%) had brain MRI abnormalities. Fifteen patients (21%) had recent cerebral infarctions in diffusion-weighted imaging sequences (Fig. 1A); these were located in the infratentorial areas (7/15), frontal lobe (3/15), parietal-occipital lobe (2/15), basal ganglia (2/15), or corpus callosum (1/15). All 44 patients with abnormal MRI showed WMH (Fig. 1B), which were located in the parietal-occipital lobe (37/44), frontal lobe (35/44), temporal lobe (16/44), basal ganglia (15/44), or infratentorial areas (6/44). Twenty-four patients (55%) presented lacunar infarctions (Fig. 1C), which were mostly located in the basal ganglia, coronary radiata, or infratentorial areas. CMB were noted in 13 of the 39 patients (33%) who underwent susceptibility-weighted imaging (Fig. 1D); these were mostly located in the basal ganglia (Fig. 2). Furthermore, 37 patients (52%) had cerebral atrophy, and thalamic hypersignal was present in five patients (7%).

#### CVSD scores

Of the 71 patients, the ranges (medians) of CVSD-related scores were as follows: ARWMC, 2–10 (4.00); Fazekas, 1–4 (2.00); EPVS, 0.25–2 (1.00); MARS, 0–2 (0); lacunar infarcts, 0–1 (0); GCA, 0–9.75 (4.00); SVDS, 1–3 (1.00) (Supplementary Table 3). Compared with the healthy controls (Supplementary Table 2), patients with Fabry disease had significantly different ARWMC (*p* < 0.001), Fazekas (*p* < 0.001), EPVS (*p* < 0.05), MARS (*p* < 0.05), lacunar infarction (*p* < 0.001), SVDS (*p* < 0.001), and GCA (*p* < 0.0001) scores. By contrast, the pulvinar sign was not significantly different between controls and patients (*p* = 0.05).

#### Subgroups analysis for CNS symptoms and MRI scores

##### Sex

The onset age (median and interquartile range) of the 46 males was 9 (7, 12) years old; this was significantly younger than that of the 25 females (18 [7, 33.5] years old; *p* = 0.03). The plasma Lyso-Gb3 (median and interquartile range) of male patients (80.5 [40.6, 97.3] ng/mL) was significantly higher than that of female patients (5.5 [3.1, 9.6] ng/mL; *p* < 0.001). There were no significant differences between male and female patients in the frequency of CNS symptoms or in any CSVD assessment scale (including lacunar infarction, WML, EPVS, CMB, SVDs, and GCA) (Table 1).

**Table 1:** Gender subgroups analysis in CNS symptoms and MRI scores

	Female	Male	P-value
No	25	46	
Onset-age(year-old)	18 (7, 33.5)	9 (7, 12)	0.03*
α-GalA	4.1 (2.15, 18.2)	0.46 (0.28, 0.8)	< 0.001*
Lyso-Gb3	5.5 (3.1, 9.6)	80.5 (40.6, 97.3)	< 0.001*
*GLA* truncated mutation	36.0%	45.7%	0.463
Smoke	0.0%	28.3%	0.003*
HT	28.0%	28.3%	> 0.999
DM	8.0%	0.0%	0.121
Hyperlipidemia	28.0%	21.7%	0.572
Hyper-HCY	8.0%	26.1%	0.116
AF	8.0%	2.2%	0.282
Headache	32.0%	10.9%	0.051
Dizziness	56.0%	52.2%	0.807
Tinnitus	28.0%	41.3%	0.311
Hearing loss	16.0%	30.4%	0.256
Vision loss	4.0%	17.4%	0.146
Cognitive decline	24.0%	21.7%	> 0.999
Depression	20.0%	15.2%	0.742
Pulvinar Sign	0.0%	10.9%	0.154
ARWML	6 (2, 10)	4 (2, 9.25)	0.452
Fazekas	2 (1, 2)	1 (1, 2)	0.277
EPVS	1 (0, 2)	1 (1, 2)	0.111
MARS	0 (0, 0)	0 (0, 2)	0.247
Lacuna	0 (0, 0.5)	0 (0, 1)	0.402
SVDS	1 (0, 1.25)	1 (1, 3)	0.143
GCA	6 (0, 9)	4 (0, 11)	0.822

*p < 0.05

##### Brain MRI

The onset-ages of the 44 patients with abnormal brain MRI were older than those of patients with normal brain MRI (*p* < 0.05). Additionally, the frequencies of dizziness (65.9%) and cognitive decline (34.1%) were higher in patients with abnormal brain MRI than in those with normal brain MRI (*p* < 0.05). There were no significant differences between the two groups in any other clinical characteristics or risk factors of CSVD (*p* > 0.05) (Table 2).

**Table 2:** Imaing subgroups analysis in CNS symptoms and risk factors of CSVD

	Abnormal bMRI	Normal bMRI	P-value
No	44	27	
Gender(M%)	65.9%	63.0%	> 0.999
Onset-age(year-old)	10 (7, 27.5)	7 (6.3, 11.8)	0.042*
Examination age	39 (30.8, 50.3)	26 (19.5, 30)	< 0.001*
Interval (year)	22.5 (12, 32)	12.5 (9,3, 20.5)	0.014*
α-GalA	0.7 (0.3, 3.1)	1 (0.4, 2.2)	0.673
Lyso-Gb3	43.3 (9.4,88.3)	32.5 (5.2, 79.7)	0.512
*GLA* truncated mutation	50.0%	29.6%	0.137
Smoke	22.7%	11.1%	0.344
HT	36.4%	14.8%	0.061
DM	4.5%	0.0%	0.522
Hyperlipidemia	31.8%	11.1%	0.084
Hyper-HCY	25.0%	11.1%	0.222
AF	4.5%	3.7%	> 0.999
Headache	22.7%	11.1%	0.344
Dizziness	65.9%	33.3%	0.014*
Tinnitus	45.5%	22.2%	0.075
Hearing loss	31.8%	14.8%	0.161
Vision loss	15.9%	7.4%	0.467
Cognitive decline	34.1%	3.7%	0.003*
Depression	22.7%	7.4%	0.115

*p < 0.05

##### Classic and non-classic types

There were 53 patients with classic-type Fabry disease (44 males and 9 females) and 18 patients with non-classic Fabry disease (2 males and 16 females). The SVDS scores (median and interquartile range) of the classic type group (1.5 [1, 3]) were higher than those of the non-classic type group (1 [0, 1]; *p* < 0.05). There were no significant differences between the two groups in any other CSVD assessment scale (Table 3).

**Table 3:** Imaging analysis between classic and non-classic type

	Classic type	Non-classic type	P-value
No	53	18	
Interval (year)	20 (11.5, 31)	9 (3, 24.5)	0.06
ARWML	5 (2, 10)	2 (0, 8.5)	0.282
Fazekas	1 (1, 2)	1 (0, 2)	0.615
EPVS	1 (1, 2)	0.5 (0, 2)	0.135
MARS	0 (0, 2)	0 (0, 0)	0.298
Lacuna	0 (0, 1)	0 (0, 0)	0.127
SVDS	1.5 (1, 3)	1 (0, 1)	0.025*
GCA	5 (0, 11)	0 (0, 7)	0.166
Pulvinar Sign	7.5%	5.6%	> 0.999

*p < 0.05

##### Clinical events

Using the severe clinical events criteria, 48 and 23 patients were classified into the mild and severe clinical phenotype groups, respectively. There were significant differences between the two groups in the pulvinar sign as well as in ARWML, Fazekas, EPVS, lacunar infarction, SVDS, and GCA scores (*p* < 0.05). However, there was no significant difference between the two groups in MARS scores (p = 0.056) (Table 4). Moreover, multivariate logistic regression analysis indicated that the higher ARWML scores were a risk factor for severe clinical events (b = 0.360, p = 0.012).

**Table 4:** Imaging analysis between mild and severe clinical events type

	Mild clinical events	Severe clinical events	P-value
No	48	23	
Interval (year)	14 (9, 23)	25 (19, 34)	0.002
ARWML	2 (1,6.75)	10 (6, 14)	< 0.001
Fazekas	1 (0, 2)	2 (2, 3)	< 0.001
EPVS	1 (0, 2)	2 (1, 2)	0.008
MARS	0 (0, 0)	0 (0, 2)	0.056
Lacunar infarct	0 (0, 0)	1 (0, 3)	< 0.001
SVDS	1 (0, 1)	3 (1, 3)	< 0.001
GCA	0 (0, 6)	8 (4.5, 13)	< 0.001
Pulvinar Sign	2.10%	17.40%	0.035

##### Genotype

Thirty patients had *GLA* truncated mutations and 41 patients had *GLA* non-truncated mutations. In brain MRI, the EPVS scores (median and interquartile range) of patients with truncated mutations (2 [1, 2]) were higher than those of patients with non-truncated mutations (1 [0, 2]; *p* = 0.013). However, there were no significant differences between the two groups in any clinical characteristics or any other imaging indicators (*p* > 0.05) (Supplementary Table 4).

#### Correlations between brain MRI scores and disease burden or biomarkers

We analyzed the correlations between imaging indicators and α-Gal A activity, Lyso-Gb3, or MSSI scores. There were significant correlations between α-Gal A activity and SVDS scores (Spearman’s *r* =  − 0.375, *p* < 0.05). There were also significant correlations between Lyso-Gb3 and SVDS (Spearman's *r* = 0.536, *p* < 0.01, EPVS (Spearman’s *r* = 0.298, *p* < 0.05), and MARS (Spearman’s *r* = 0.380, *p* < 0.05) scores. Additionally, MSSI scores were significantly correlated with all CVSD scores (i.e., ARWML, Fazekas, EPVS, MARS, lacunar infarcts, SVDS, and GCA scores) (Table 5).

**Table 5:** Correlations between brain MRI with disease burden and biomarkers

Spearman/Pearson	ARWML	Fazekas	EPVS	MARS	Lacuna	SVDS	GCA	Pulvinar Sign
Lyso-Gb3								
r	0.129	0.096	0.298^*^	0.380^*^	0.086	0.536^**^	0.142	– 0.080
P	0.362	0.499	0.032	0.035	0.542	0.002	0.317	0.574
α-GalA								
r	0.111	0.184	– 0.114	– 0.263	-0.038	– 0.375*	– 0.030	0.015
P	0.360	0.128	0.349	0.101	0.755	0.017	0.808	0.901
MSSI								
r	0.365^**^	0.324^**^	0.282^*^	0.334^*^	0.321^**^	0.521^**^	0.355^**^	0.105
P	0.002	0.007	0.020	0.037	0.008	0.001	0.003	0.395

*p < 0.05; **p < 0.01

#### Brain MRI follow-up and its correlations with disease burden and biomarkers

Twenty-three patients were followed up with brain MRI. The follow-up time(median and interquartile range) was 2.92 (2.21, 5.71) years. The change rates of ARWMC (0.5 [0.26, 1.56]/year) and GCA (0.87 [0.05, 1.94]/year) scores were the markedly increased indicators (Supplementary Table 5). We then analyzed the correlations between imaging follow-up indicators and α-Gal A activity, Lyso-Gb3, or MSSI scores. There were significant correlations between α-Gal A activity and the median change rate of MARS scores (Pearson’s *r* =  − 0.870, *p* < 0.05), and between MSSI scores and the median change rate of lacunar infarction (Pearson’s r = 0.535, *p* < 0.05 ) and GCA scores (Pearson’s *r* = 0.537, *p* < 0.05) (Table 6).

**Table 6:** Correlations between brain MRI follow-up with disease burden and biomarkers

Spearman/Pearson	▲ARWML	▲Fazekas	▲EPVS	▲MARS	▲Lacuna	▲SVDS	▲GCA
Lyso-Gb3							
r	– 0.022	0.153	0.325	0.058	– 0.264	– 0.259	0.290
P	0.940	0.601	0.256	0.913	0.361	0.574	0.315
α-GalA							
r	– 0.320	– 0.315	0.278	.870^*^	0.318	0.074	0.012
P	0.227	0.234	0.298	0.024	0.230	0.875	0.965
MSSI							
r	0.233	– 0.043	0.348	0.691	0.535^*^	0.222	.537^*^
P	0.385	0.874	0.187	0.128	0.033	0.632	0.032

## Discussion

In our cohort, 16 patients (22.5%) with Fabry disease experienced ischemic stroke. The onset age of first stroke was around 40 years, which is in accordance with the average onset age in the Fabry Registry [19]. Aside from stroke episodes, we also confirmed nonspecific headache, dizziness, and neuropsychiatric dysfunction in approximately half of all patients, similar to a previous report [20]. Ear abnormalities, including hearing loss, tinnitus, and vertigo, are frequently noted in patients with Fabry disease, and hearing loss is correlated with vascular damage and neuropathy [21]. Notably, in the present study, the rate of abnormal MRI findings did not reflect the rate of CNS symptoms; this finding indicates that some symptoms may not only be caused by brain structural alterations, but also by peripheral nerve system dysfunction or the burden of chronic multiorgan dysfunction. [22]

Our results indicated that approximately half of all patients were affected on brain MRI. In a previous study by Reisin et al., the average affected age was 27 years old. [5] We also found that abnormal MRI tended to present at an older age, but appeared as early as 7 years old. Brain MRI in our patients showed progressive lacunar infarctions, WMH, CMB, and decreased brain volume, which are all characteristic of CSVD. Although previous studies have reported these imaging characteristics in Fabry disease, our study presented the burden of these lesions through detailed CSVD scoring, and described their probable predilections for specific brain regions. The incidences of lacunar infarctions and WMH in our group were 55% and 62%, respectively. Fazekas reported a similar WMH frequency, of 46.4%, in young patients with symptomatic stroke of all causes [23]. Furthermore, both our study and previous studies [24] revealed similar frequencies of WMH in male and female patients, which indicates that there is no difference in CSVD between male and female patients. This finding is similar to the incidence of cardiac events but unlike that of kidney events, where male patients are more affected. Previous literature suggests that WMH are generally sparse and unspecific [24]. In our study, some cases showed a high WMH load with periventricular and diffuse leukoencephalopathy. CMB are considered more common; they are reportedly observed in 11–30% of patients with Fabry disease, independently of symptoms of CNS involvement [8]. We therefore demonstrated a relatively high prevalence of patients with CMB (33%), but most of our cohort had mild CMB (only four male patients had multiple CMB) compared with sporadic CVSD, which shows deeper and infratentorial lesions such as WML, CMB, and lacunar infarctions [25]. In our study, five male patients (7%) showed a pulvinar sign; this is lower than the mean prevalence (23.9%) reported in previous studies, although previous studies also reported a much higher prevalence of the pulvinar sign in men than in women [26]. Hyperintensity in the pulvinar likely reflects selectively increased cerebral blood flow in the thalamus; however, there is no correlation between stroke occurrence and the pulvinar sign [27]. In our longitudinal follow-up cohort, there was a marked exacerbation of WMH and cerebral atrophy in the brain MRI of patients with Fabry disease. That is, our long-term follow-up revealed that brain MRI evolution in Fabry disease reflects mainly white matter and cerebral atrophy changes, which is different from what is observed in sporadic CSVD. [28]

Although male patients had much lower α-Gal A activity and higher Lyso-Gb3 than female patients in the present study, there was no observed impact of sex on CNS symptoms or brain MRI lesions; this is similar to what has been reported in previous studies [24, 29]. Moreover, our study indicated that although phenotypic differences exist between male and female patients, the extent of CNS involvement may not differ between the sexes; prevention of stroke in female patients should thus be given equal attention to that in male patients. In addition, SVDS scores were higher in patients with classic-type Fabry disease and more severe clinical phenotypes. This may be because of multi-system involvement, including that of the heart and kidney, which also leads to increased burden on cerebral small vessels. Fabry disease is clinically heterogeneous, and in women its clinical severity has recently been linked to the skewing of X-inactivation [30]. To date, nearly 1000 different *GLA* variants have been reported. Nonsense and frameshift variants are often considered to be pathogenic with a classic phenotype, while missense and splicing substitution variants may be pathogenic with non-classic or late-onset phenotypes [12]. However, our study and previous investigations were unable to confirm a specific and definite relationship between individual *GLA* variant types and CNS symptoms or neuroimaging severity. This finding suggests that whether or not the *GLA* encoded-protein structure is changed does not affect the CNS neuroimaging phenotype, possibly because the site of missense mutations may be located near the active site of the enzyme.

Both α-Gal A activity and plasma Lyso-Gb3 have been analyzed as diagnostic biomarkers for classic and late-onset Fabry disease in male and female patients; these markers reflect disease activity and severity, at least in some patients [2]. Previous studies have indicated that Lyso-Gb3 is related to kidney and cardiac involvement; however, a clear correlation between Lyso-Gb3 concentrations and clinical CNS manifestations has not yet been demonstrated. Our analysis revealed that possible biomarkers of Fabry disease (e.g., α-Gal A activity and Lyso-Gb3) and markers of disease burden (e.g., MSSI) were closely correlated with the CVSD total burden. Furthermore, WMH correlated with multiple organ burden in Fabry disease (as MSSI), which suggests that CNS progression is consistent with general progression. In a previous study, endothelial nitric oxide synthase uncoupling has been detected in the presence of deficiency; this may be a potential explanation for altered vascular function, supporting the critical role of vascular dysfunction in the pathogenesis of brain perfusion changes, because Gb3 accumulation leads to the non-arteriosclerotic injury of vascular endothelial cells [31]. Brain MRI lesions and WMH burden increase with age and can even precede the onset of neurological symptoms in Fabry disease. [29] Moreover, the presence of severe WMH is an indicator of stroke recurrence up to 5 years after a first-ever ischemic stroke [7]. The results of our follow-up brain MRI study suggest that the progression of CMB, lacunar infaction, and cerebral atrophy in the brain might reflect disease activity and severity as a whole in Fabry disease.

In conclusion, our results support brain MRI as not only a tool to show brain structure, but also as a valuable indicator of disease burden as a whole in Fabry disease. Moreover, the close relationship between brain MRI features and the biomarkers of Fabry disease may reveal further mechanisms of cerebral small vessel damage. One major limitation of our study was that the MRI protocol was not uniform among centers, some sequences that are specific for certain cerebral abnormalities, like hemosiderin deposits, were not available for all patients. In addition, patients with Fabry disease presenting stroke and reported cognitive problems more likely to be seen in the neurology department and underwent brain MRI, there may be some degree of selection bias. In the future, with the rapid progress of imaging technology and its use in sporadic and other hereditary CVSD [32, 33], high-resolution 7 T MRI is expected to be used in patients with Fabry disease to evaluate cerebral lesions and cerebral small vascular changes. In addition, the long-term follow-up of brain MRI after enzyme replacement therapy will be helpful to optimize potential interventions for CNS lesions, and to better understand the mechanisms of brain dysfunction in Fabry disease.

### Supplementary Information

Below is the link to the electronic supplementary material.

Supplementary file1 (PDF 1426 KB)

## Data Availability

The authors confirm that the data supporting the findings of this study are available within the article and its supplementary material.
