# Supplementary material for: Brain MRI correlations with disease burden and biomarkers in Fabry disease
Source: J Neurol. 2023 Jun 25;270(10):4939–48. doi: 10.1007/s00415-023-11826-8 (PMC10511580; doi:10.1007/s00415-023-11826-8)
Supplement: Supplementary file 1 — Supplementary file1 (PDF 1426 KB) [file 415_2023_11826_MOESM1_ESM.pdf]

Supplement table 1. Demographic data of patients with Fabry disease

Family	No.	Gender	α-GalA (%)	Lyso-Gb3 (ng/ml)	*GLA* mutation	Onset age (year-old)	CNS manifestations	Risk factors	MSSI	Classic type	Clinical event
F1	P1	M	2%	97.3	c.929T>G p.(Leu310Arg)	7	Stroke, Diziness, Tinnitus, Hearing loss	(-)	45	(+)	(+)
F2	P2	F	49%	20.26	c.1188_1208dup p.(Phe396_Arg402dup)	6	Stroke, Diziness, Hearing loss, Cognitive decline,Depressi	HT, Hyperlipidemia , Hyper-HCY	48	(+)	(+)
F2	P3	F	91%	10.84		6	Stroke, Headache, Diziness, Tinnitus, Hearing	Hyper-HCY	24	(+)	(+)
F3	P4	M	2%	32.47	c.983G>A p.(Gly32	10	(-)	(-)	19	(+)	(-)
F3	P5	F	14%	3.08		7	(-)	(-)	11	(-)	(-)
F3	P6	M	2%	101.69		10	(-)	(-)	17	(+)	(-)
F3	P7	F	13%	3.31		10	Headache, Diziness	HT, Hyperlipide	18	(-)	(-)
F4	P8	M	1%	103.84	c.901C>T p.(Arg301*)	7	Diziness, Tinnitus, Vision loss, Cognitive decline	(-)	23	(+)	(-)
F5	P9	M	1%	ND	c.1020G>A p.(Trp34	13	(-)	(-)	8	(+)	(-)
F6	P10*	M	24%	ND	c.1033_1034del p.(Ser345Argfs*29)	29	Stroke, Diziness	Hyperlipidemia	32	(+)	(+)
F7	P11	F	Nor.	ND	c.901C>T p.(Arg30	11	Diziness	(-)	11	(+)	(-)
F8	P12	M	2%	87.2	c.680G>A p.(Arg227Gln)	7	Stroke, Headache, Diziness,	HT, Hyperlipidemia ,	48	(+)	(+)
F9	P13	M	3%	127.34	Duplication (exon6)	7	(-)	(-)	11	(+)	(-)
F10	P14	M	1%	ND	c.485G>A p.(Trp162*)	7	Diziness, Tinnitus, Hearing loss	(-)	24	(+)	(-)
F11	P15	M	2%	89.18	c.916C>T p.(Gln306*)	10	Diziness, Tinnitus, Hearing loss	(-)	20	(+)	(-)
F12	P16	M	3%	65.56	c.466G>A p.(Ala156Thr)	10	Stroke, Diziness, Vision loss	(-)	11	(+)	(+)
F13	P17	M	1%	81.23	c.59_72del p.(Ala20Glyfs*6)	9	Diziness, Tinnitus, Hearing loss	(-)	19	(+)	(-)
F14	P18	M	5%	51.71	c.463G>C p.(Asp155His)	7	Stroke, Headache, Diziness, Hearing loss,Cognitive	Hyperlipidemia	34	(+)	(+)
F14	P19	F	33%	2.99		7	Headache, Cognitive	HT	18	(+)	(-)
F15	P20	M	5%	ND	c.758T>C p.(Ile253Thr)	8	Stroke, Diziness, Cognitive decline	HT	42	(+)	(+)
F15	P21	M	3%	11.05		27	(-)	HT, Hyperlipidemia ,	31	(+)	(-)
F15	P22	F	93%	ND		5	Headache,	(-)	5	(-)	(-)
F16	P23	M	5%	ND	c.728T>C p.(Leu24	7	Dizziness	(-)	ND	(+)	(-)
F16	P24	F	Nor.	ND		7	(-)	(-)	ND	(+)	(-)
F17	P25	M	1%	89.75	c.680G>A p.(Arg227Gln)	29	Diziness, Tinnitus, Hearing loss, Cognitive decline,Depressi	HT, Hyperlipidemia	59	(+)	(+)
F17	P26	F	21%	3.1		22	Tremor	(-)	2	(-)	(-)
F18	P27	M	4%	55.34	c.803_806del p.(Leu268*)	9	Tinnitus, Vision loss, Cognitive decline,Depressi	HT, Hyperlipidemia	23	(+)	(-)
F18	P28	F	6%	7.95		31	(-)	(-)	13	(-)	(-)
F18	P29	M	3%	105.12		7	(-)	(-)	10	(+)	(-)
F19	P30	F	Nor.	ND	large intragenic deletions	5	Diziness, Cognitive	Hyperlipidemia	28	(+)	(+)
F19	P31	M	3%	200		9	Diziness, Tinnitus, Hearing loss	(-)	33	(+)	(+)
F20	P32	M	6%	ND	c.605G>T p.(Cys20	6	(-)	(-)	10	(+)	(-)
F20	P33	M	1%	ND		7	Diziness	(-)	36	(+)	(-)
F21	P34	F	10%	8.25	c.1024C>T p.(Arg34	50	Diziness	HT	6	(-)	(-)
F21	P35	M	1%	81.14		7	Stroke,	HT, Hyper-	38	(+)	(+)
F22	P36	M	14%	ND	c.453C>A p.(Tyr151	5	Tinnitus, Hearing	(-)	33	(+)	(+)
F23	P37	M	8%	ND	c.815A>G p.(Asn27	5	Diziness, Tinnitus	(-)	20	(+)	(-)
F24	P38	M	5%	97.8	c.658C>T p.(Arg220*)	7	Diziness, Tinnitus, Vision loss	HT, Hyper-HCY	13	(+)	(-)
F24	P39	F	Nor.	9.37		18	Diziness, Tinnitus, Cognitive decline	(-)	7	(-)	(-)
F25	P40	M	2%	16.25	c.973G>A p.(Gly32	12	(-)	(-)	19	(+)	(-)
F26	P41	M	2%	80.17	large intragenic deletions	7	(-)	(-)	12	(+)	(-)

Supplement table 1 (Continued)

Family	No.	Gender	α-GalA (%)	Lyso-Gb3 (ng/ml)	*GLA* mutation	Onset age (year-old)	CNS manifestations	Risk factors	MSSI	Classic type	Clinical event
					deletions						
F27	P42	F	47%	5.79	c.167G>T p.(Cys56	32	(-)	(-)	6	(-)	(-)
F28	P43	M	1%	88.31	c.1094dup p.(Tyr365 *)	10	Stroke, Headache, Diziness, Tinnitus, Vision loss,	(-)	20	(+)	(+)
F29	P44	M	4%	ND	c.132G>A p.	9	Stroke, Diziness	(-)	12	(+)	(+)
F30	P45	M	10%	ND	c.440G>A p.(Gly14	9	Hearing loss	Hyper-HCY	6	(+)	(-)
F30	P46	F	12%	5.2		12	Diziness, Tinnitus, Hearing loss	AF	9	(-)	(-)
F31	P47	M	1%	97.3	c.511G>T p.(Gly17	12	Tinnitus, Hearing	Hyper-HCY	15	(+)	(-)
F32	P48	M	3%	18.64	c.511G>C p.(Gly17 1Cys)	15	(-)	HT, Hyperlipide mia ,	31	(+)	(+)
F33	P49	M	3%	88.41	c.548-2A>G p.splicin g	7	Stroke, Headache,	Hyperlipide mia	22	(+)	(+)
F34	P50	F	9%	5.1	c.334C>T p.(Arg11 2Cys)	40	Headache, Diziness, Tinnitus	(-)	6	(-)	(-)
F34	P51	M	1%	43.3		10	Tinnitus	Hyper-HCY	28	(+)	(+)
F35	P52	F	17%	1.27	c.644A>G p.(Asn21 5Ser)	70	Stroke, Headache, Diziness,	HT	13	(+)	(+)
F36	P53	M	2%	ND	c.284G>A p. (Trp95*)	7	Headache, Diziness, Tinnitus,Cognitiv	HT, Hyperlipide mia, Hyper-	22	(+)	(-)
F36	P54	F	Nor.	ND		10	Headache, Diziness, Tinnitus,Hearing	HT,DM	10	(-)	(-)
F37	P55	M	1%	61.32	c.782G>A p.(Gly26	6	Vision loss	HT	6	(+)	(-)
F38	P56	M	2%	79.19	c.779G>C p.(Gly26	6	(-)	(-)	17	(+)	(-)
F39	P57	F	Nor.	5.2	c.506T>C p.(Phe16 9Ser)	35	(-)	HT, Hyperlipide	ND	(-)	(-)
F40	P58	F	21%	12.51	c.266T>G p.(Leu89	29	(-)	Hyperlipide	12	(+)	(-)
F41	P59	M	2%	20.74	c.109G>A p.(Ala37T hr)	33	Tinnitus,Hearing loss, Vision loss	HT, Hyper-HCY	31	(+)	(+)
F42	P60	M	4%	80.79	c.146G>T p.(Arg49 Leu)	8	Diziness, Tinnitus, Hearing loss,Depression	(-)	22	(+)	(-)
F43	P61	M	11%	9.58	c.634C>T p.(Gln21 2*)	12	Stroke, Diziness, Cognitive decline	HT, AF	27	(+)	(+)
F44	P62	F	11%	10.43	c.680G>A p.(Arg22	18	Depression	Hyperlipide	13	(+)	(-)
F45	P63	M	2%	71.38	c.132G>T p.(Trp44	6	Hearing loss	(-)	19	(+)	(-)
F46	P64	M	4%	44.92	c.511G>C p.(Gly17 1Arg)	12	Stroke, Diziness, Tinnitus, Hearing loss,Cognitive decline	HT, Hyperlipide mia , Hyper-HCY	46	(+)	(+)
F46	P65	F	6%	1.43		30	(-)	(-)	1	(-)	(-)
F47	P66	F	18%	1.17	c.874G>C p.(Ala292	50	Diziness, Tinnitus	DM	3	(-)	(-)
F48	P67	M	5%	5.88	c.640- p.splicin	NS	(-)	(-)	0	(-)	(-)
F49	P68	F	26%	6.87	c.613C>A p.(Pro20 5Thr)	51	Diziness, Cognitive decline	Hyperlipide mia	4	(-)	(-)
F50	P69	M	7%	3.43	c.640- p.splicin	37	Tinnitus,	(-)	8	(-)	(-)
F50	P70	F	ND	1.69		4	Headache	(-)	6	(-)	(-)
F51	P71	M	3%	129.25	c.275A>T p.Asp92	12	Stroke, Diziness	Hyper-HCY	35	(+)	(+)

*: died at the age of 34; M:Male; F:Female; NS:No symptom; ND: Not done; Nor.:Normal; HT: Hypertension; DM:Diabetes;Hyper-HCY: Hyperhomocysteinemia; AF:Atrial fibrillation

Supplement table 2 . The CVSD scores of healthy control

No.	Gender	Examination age	ARWML	Fazekas	EPVS	MARS	Lacuna	SVDS	GCA	Pulvinar Sign
C1	1	7	0	0	1	0	0	0	0	0
C2	1	16	1	1	0	0	0	0	0	0
C3	1	16	0	0	0	0	0	0	0	0
C4	1	17	0	0	1	0	0	0	0	0
C5	0	18	0	0	0	0	0	0	0	0
C6	1	19	0	1	1	0	0	0	0	0
C7	1	19	2	1	0	0	0	0	2	0
C8	1	20	0	0	2	ND	0	ND	0	0
C9	0	21	0	0	0	ND	0	ND	0	0
C10	1	22	0	1	0	ND	0	ND	0	0
C11	1	22	0	0	0	0	0	0	0	0
C12	1	24	0	0	0	ND	0	ND	0	0
C13	1	24	0	0	0	0	0	0	0	0
C14	1	25	0	0	1	ND	0	ND	0	0
C15	1	26	0	1	1	ND	0	ND	0	0
C16	1	26	0	0	1	0	1	0	0	0
C17	1	26	0	0	2	ND	0	ND	2	0
C18	1	26	0	0	1	ND	0	ND	0	0
C19	1	27	0	1	1	0	0	0	0	0
C20	1	28	1	0	2	ND	0	ND	2	0
C21	0	29	0	0	0	0	0	0	0	0
C22	0	29	0	1	2	ND	0	ND	0	0
C23	1	30	2	1	0	0	0	0	0	0
C24	0	30	0	0	0	ND	0	ND	0	0
C25	1	30	1	0	0	0	0	0	0	0
C26	1	31	0	0	1	ND	0	ND	2	0
C27	1	32	0	0	1	0	0	0	0	0
C28	0	33	0	0	1	ND	0	ND	0	0
C29	1	34	0	1	0	0	0	0	0	0
C30	1	35	0	0	2	ND	0	ND	0	0
C31	1	35	0	1	0	ND	0	ND	0	0
C32	1	38	0	1	1	0	0	0	0	0
C33	1	38	0	0	0	0	0	0	0	0
C34	0	39	0	1	1	ND	0	ND	2	0
C35	1	39	0	0	1	0	0	0	0	0
C36	0	41	0	0	0	ND	0	ND	0	0
C37	1	42	0	0	1	0	0	0	2	0
C38	1	43	0	1	1	0	0	0	0	0
C39	0	43	0	0	1	ND	0	ND	0	0
C40	1	43	0	0	1	ND	0	ND	0	0
C41	1	43	0	0	1	0	0	0	0	0
C42	1	44	0	0	0	ND	0	ND	0	0
C43	0	45	0	1	0	0	0	0	0	0
C44	1	47	2	1	0	0	0	0	0	0
C45	0	49	0	1	2	ND	0	ND	0	0
C46	1	49	0	1	1	ND	0	ND	0	0
C47	1	50	1	0	0	ND	0	ND	0	0
C48	1	51	0	1	0	0	0	0	0	0
C49	0	54	0	1	0	0	0	0	0	0
C50	0	57	2	1	0	ND	0	ND	0	0
C51	0	62	0	1	1	ND	0	ND	0	0
C52	0	70	0	1	1	ND	0	ND	0	0
C53	0	77	0	0	0	0	0	0	0	0

Supplement table 3 . The CVSD scores of Fabry disease

Family	No.	Gender	Examination age(year-old)	ARWML	Fazekas	EPVS	MARS	Lacuna	SVDS	GCA	Pulvinar Sign
F1	P1	M	30	16	3	1	2	1	3	11	(-)
F2	P2	F	54	10	2	3	5	3	3	8	(-)
F2	P3	F	29	19	3	4	0	6	2	16	(-)
F3	P4	M	25	2	0	0	ND	0	ND	0	(-)
F3	P5	F	30	2	1	0	ND	0	ND	0	(-)
F3	P6	M	28	2	0	2	ND	0	ND	0	(-)
F3	P7	F	57	18	3	2	ND	3	ND	15	(-)
F4	P8	M	43	4	1	2	ND	0	ND	6	(-)
F5	P9	M	24	0	0	2	ND	0	ND	2	(-)
F6	P10	M	32	16	3	2	ND	2	ND	17	(+)
F7	P11	F	21	2	1	1	ND	0	ND	0	(-)
F8	P12	M	31	6	2	0	1	0	2	6	(-)
F9	P13	M	20	2	1	0	ND	0	ND	0	(-)
F10	P14	M	38	5	1	1	0	0	1	5	(-)
F11	P15	M	27	4	1	2	4	2	3	0	(-)
F12	P16	M	26	14	3	3	ND	3	ND	11	(+)
F13	P17	M	21	8	2	3	0	0	2	14	(-)
F14	P18	M	36	6	2	2	0	1	1	5	(-)
F14	P19	F	39	6	2	0	ND	1	ND	6	(-)
F15	P20	M	50	14	3	2	4	3	3	7	(+)
F15	P21	M	47	4	1	1	0	0	1	13	(-)
F15	P22	F	29	2	1	1	ND	0	ND	0	(-)
F16	P23	M	43	2	1	1	0	0	0	0	(-)
F16	P24	F	18	2	1	0	0	0	0	0	(-)
F17	P25	M	51	14	3	2	0	2	3	13	(-)
F17	P26	F	23	0	0	0	0	0	0	0	(-)
F18	P27	M	24	4	1	0	0	0	1	2	(-)
F18	P28	F	45	8	2	0	0	0	1	4	(-)
F18	P29	M	39	8	2	2	2	4	3	11	(-)
F19	P30	F	62	14	3	1	ND	0	ND	13	(-)
F19	P31	M	34	4	1	2	0	0	1	4	(-)
F20	P32	M	7	0	0	1	0	0	0	0	(-)
F20	P33	M	26	0	0	1	0	0	0	0	(-)
F21	P34	F	65	16	3	2	ND	2	ND	12	(-)
F21	P35	M	37	2	1	3	2	1	3	8	(-)
F22	P36	M	26	2	1	1	ND	0	ND	0	(-)
F23	P37	M	15	2	1	1	0	0	1	0	(-)
F24	P38	M	16	2	1	1	0	0	1	2	(-)
F24	P39	F	43	14	3	2	ND	0	ND	6	(-)
F25	P40	M	22	10	2	2	2	0	2	17	(-)
F26	P41	M	17	2	1	1	ND	0	ND	0	(-)
F27	P42	F	34	8	2	2	0	0	1	6	(-)
F28	P43	M	42	17	3	3	4	4	4	18	(-)
F29	P44	M	43	12	2	2	0	3	3	13	(-)
F30	P45	M	16	0	0	0	0	0	0	0	(-)
F30	P46	F	57	0	0	0	ND	0	ND	0	(-)
F31	P47	M	17	2	1	0	2	0	2	0	(-)
F32	P48	M	32	5	1	1	0	0	1	0	(-)
F33	P49	M	38	10	2	1	2	1	3	13	(-)
F34	P50	F	49	0	0	0	ND	0	ND	0	(-)
F34	P51	M	22	5	1	0	0	0	1	0	(-)
F35	P52	F	77	10	2	0	0	0	1	8	(-)
F36	P53	M	26	0	0	1	ND	0	ND	2	(-)
F36	P54	F	53	10	2	2	ND	0	ND	10	(-)
F37	P55	M	19	1	1	1	ND	0	ND	0	(-)
F38	P56	M	30	0	0	1	0	0	0	9	(-)
F39	P57	F	41	2	1	1	ND	0	ND	0	(-)
F40	P58	F	49	9	2	1	ND	0	ND	8	(-)
F41	P59	M	43	8	2	1	0	2	3	4	(+)
F42	P60	M	30	8	2	2	ND	0	ND	5	(-)
F43	P61	M	53	9	2	1	ND	1	ND	4	(-)
F44	P62	F	30	2	1	1	0	0	1	0	(-)
F45	P63	M	27	2	1	0	ND	1	ND	0	(-)
F46	P64	M	69	12	2	2	3	4	4	14	(-)
F46	P65	F	33	0	0	0	0	0	0	0	(-)
F47	P66	F	57	1	1	2	ND	1	ND	6	(-)
F48	P67	M	34	0	0	0	0	0	0	0	(-)
F49	P68	F	58	7	2	0	ND	0	ND	10	(-)
F50	P69	M	38	2	1	2	1	0	1	0	(+)
F50	P70	F	7	6	2	0	0	0	1	0	(-)
F51	P71	M	45	14	3	2	ND	0	ND	17	(-)

Supplement table 4. Imaging analysis between truncated mutation and non-truncated mutation group

	Truncated mutation	Non-truncated mutation	P-value
**No.**	30	41	
**Gender(M%)**	70%	61%	0.463
**Onset-age(year-old)**	9(7, 11.5)	10(7, 28)	0.128
**α-GalA**	0.7(0.34, 3.35)	0.8(0.35, 2.77)	0.952
**Lyso-Gb3**	80.66(9.42, 95.65)	19.69(5.13, 77.24)	0.053
**Smoke**	16.7%	19.5%	>0.999
**HT**	26.7%	29.3%	>0.999
**DM**	3.3%	2.4%	>0.999
**Hyperlipidemia**	20.0%	26.8%	0.582
**Hyper-HCY**	16.7%	22.0%	0.764
**AF**	6.7%	2.4%	0.57
**Headache**	20.0%	17.1%	0.766
**Dizziness**	63.3%	46.3%	0.228
**Tinnitus**	46.7%	29.3%	0.145
**Hearing loss**	26.7%	24.4%	0.335
**Vision loss**	20.0%	7.3%	0.154
**Cognitive decline**	30.0%	17.1%	0.254
**Depression**	20.0%	14.6%	0.75
**Pulvinar Sign**	6.7%	7.3%	>0.999
**ARWML**	5.5(2, 10.5)	2(1.5, 8.5)	0.133
**Fazekas**	1.5(1, 2)	1(0.5, 2)	0.236
**EPVS**	2(1,2)	1(0, 2)	0.013*
**MARS**	0(0, 2)	0(0, 0.75)	0.234
**Lacuna**	0(0, 2)	0(0, 1)	0.289
**SVDS**	2(1,3)	1(0, 2)	0.059
**GCA**	4.5(0, 12.25)	0(0, 8.5)	0.149

Supplement table 5. Brain MRI follow-up in Fabry disease

No.	Gender	Lyso-Gb3	α-GalA	MSSI	Follow-up years	▲ARWML	▲Fazekas	▲EPVS	▲MARS	Lacuna	▲SVDS	▲GCA
**P1**	1	97.3	0.32	45	2.58	1.55	0.39	0.39	ND	0.39	ND	0.00
**P12**	1	87.2	0.35	48	7.50	1.33	0.27	0.27	ND	0.53	ND	2.09
**P15**	1	89.18	0.3	20	5.67	0.71	0.18	0.00	0.18	0.00	0.53	0.35
**P17**	1	81.23	0.24	19	11.08	0.18	0.00	0.00	0.18	0.00	0.09	0.09
**P18**	1	51.71	0.9	34	8.83	0.45	0.11	0.11	ND	0.57	ND	0.86
**P20**	1	ND	0.8	42	5.75	1.04	0.00	0.35	ND	0.00	ND	2.37
**P25**	1	89.75	0.22	59	7.58	1.45	0.13	0.26	ND	0.53	ND	6.17
**P31**	1	200	0.6	33	3.00	0.33	0.00	1.00	0.33	0.00	0.00	1.88
**P43**	1	88.31	0.1	20	2.08	4.32	0.48	0.48	0.00	0.00	0.00	0.87
**P45**	1	ND	1.8	6	2.42	0.00	0.41	0.41	ND	0.00	ND	0.00
**P47**	1	97.3	0.2	15	1.83	3.27	0.55	0.00	0.00	0.00	0.00	1.09
**P48**	1	18.64	0.52	31	1.92	2.09	1.04	0.00	ND	0.00	ND	0.67
**P49**	1	88.41	0.5	22	2.33	0.00	0.00	0.00	0.43	0.43	0.00	0.96
**P55**	1	61.32	0.2	6	2.33	0.43	0.00	0.00	ND	0.00	ND	0.00
**P60**	1	80.79	0.73	22	0.83	2.40	0.00	0.00	ND	1.20	ND	3.60
**P8**	1	103.84	0.1	23	2.00	0.50	0.50	0.00	ND	0.00	ND	2.00
**P2**	0	20.26	8.7	48	2.92	2.74	0.34	0.00	1.71	1.71	0.00	0.54
**P26**	0	3.1	3.63	2	4.00	0.00	0.00	0.00	0.00	0.00	0.05	0.89
**P3**	0	10.84	16	24	2.67	0.38	0.00	0.00	ND	0.75	ND	3.75
**P42**	0	5.79	8.3	6	4.25	0.47	0.47	0.00	ND	0.94	ND	0.91
**P5**	0	3.08	2.54	11	8.58	0.00	0.00	0.12	0.00	0.00	0.00	0.00
**P7**	0	3.31	2.37	18	5.58	0.00	0.00	0.00	ND	0.00	ND	0.00
**P70**	0	ND	ND	6	1.92	1.57	0.52	0.00	0.00	0.00	0.00	0.00
